# Contributions of aquatic organisms to understanding the vertebrate ocular lens

**DOI:** 10.3389/fopht.2026.1900415

**Published:** 2026-09-23

**Authors:** Mason Posner

**Affiliations:** Department of Biology and Toxicology, Ashland University, Ashland, OH, United States

**Keywords:** aging and regeneration, aquatic species, comparative lens biology, gene editing, lens aging and cataract, lens crystallins, lens development, lens optics

## Abstract

Aquatic species have played a vital role over the past 200 years in detailing the structure, physiology, development, and evolution of the ocular lens. As a living piece of biological “glass”, the lens must balance the optical demands of transparency and refractive power with the requirements of a metabolically active tissue. Lens researchers have leveraged unique aspects of aquatic species, such as 1) the lack of corneal refraction and the simplification of modeling aquatic lens optics, 2) the presence of external embryos for real-time observation of eye and lens development, and 3) the diversity of habitats, ecology, and genetics that support comparative studies. Discoveries across many areas of aquatic lens research contribute to understanding human blindness, tissue aging and regeneration, optical and protein design, and fundamental features of cell biology relevant to many tissues. This review highlights key contributions in these areas and points to possible future areas of discovery.

## Introduction

For more than two centuries aquatic species have provided unique opportunities to understand the function of one of the body’s most remarkable organs, the ocular lens. From early investigations into how biological lenses focus light while removing spherical aberration to modern studies using molecular tools and advanced imaging to explore lens development and regeneration, aquatic species have been central to foundational research in lens biology. The diversity of aquatic species has enabled investigation of how lenses adapt to different visual environments and has been key to detailing the molecular evolution that co-opted proteins for new functions in the lens. This review will explore the important roles aquatic species have played in describing lens evolution, biochemistry, physiology, optics and development (**Figure 1**). It will summarize current research involving aquatic models and suggest how comparative and model-based approaches can address open questions in lens biology, while looking ahead to future discoveries.

**Figure 1:**
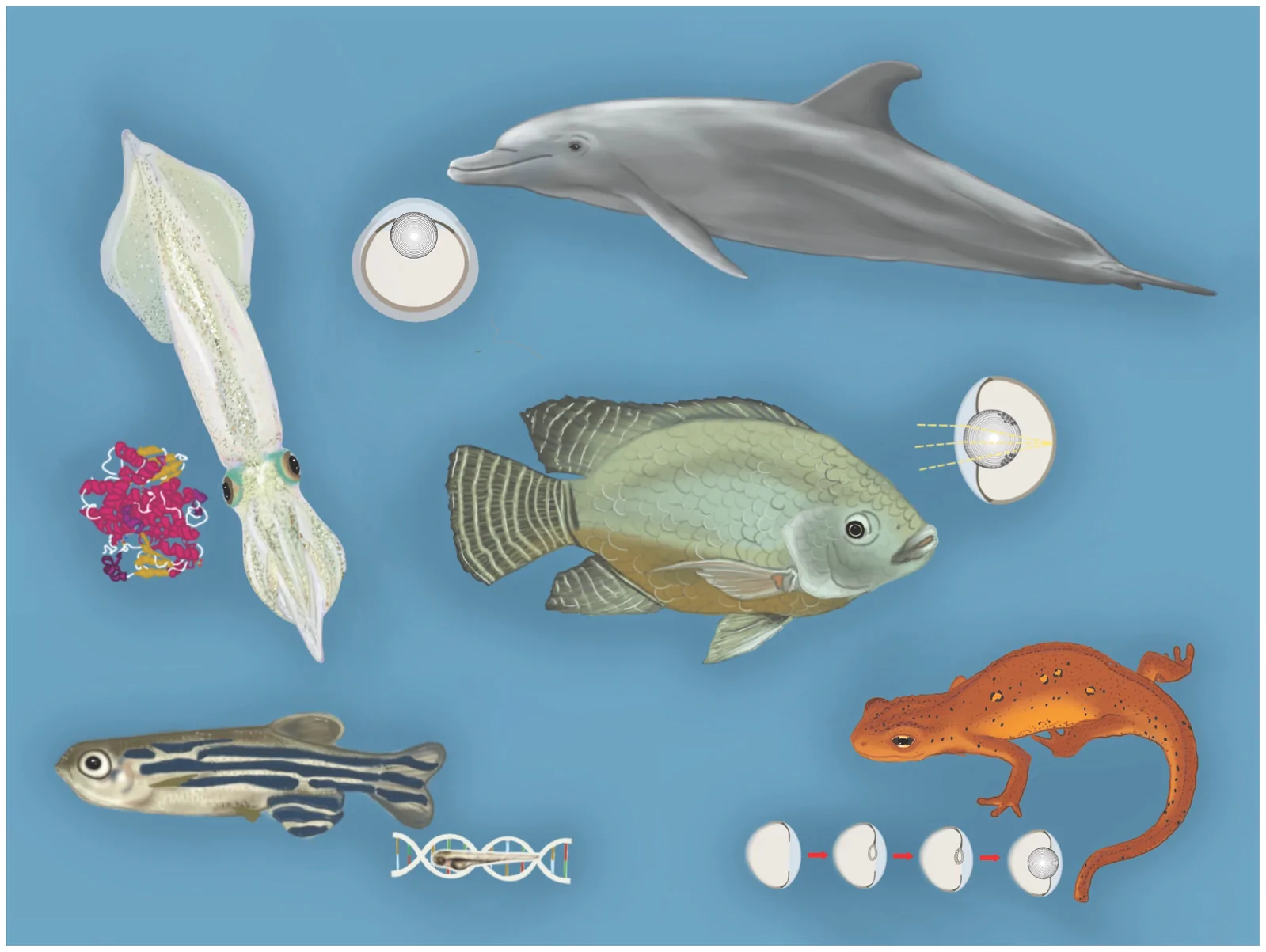
Diverse aquatic species have been used to understand lens biology. Clockwise from the upper left: protein evolution in squid; aerial and aquatic optics in cetaceans; gradient refractive index and removal of chromatic aberration in tilapia; lens regeneration in the newt; development and gene editing in zebrafish. Illustration by Marin Dixon.

Thomas Young’s foundational 1801 description of visual optics and accommodation in the human eye was closely followed by multiple studies of fish species (1, 2). Brewster (3) used polarized light to identify concentric layers in sheep, oxen, and haddock lenses. He also identified a gradient refractive index (GRIN), the change in protein density across the lens, which he hypothesized prevented spherical aberration. The most thorough early use of fish lenses to understand visual optics came from German scientist Ludwig Matthiessen. In a series of papers Matthiessen detailed the variable refractive indices of the concentric layers in the fish lens, used several fish species to demonstrate the lack of spherical aberration (aplanatic lenses) and defined Matthiessen’s ratio, showing that the focal length of the lens was approximately 2.55 times the lens radius (4, 5). Matthiessen took advantage of the dense, spherical nature of the fish lens, and the lack of refraction from the cornea in aquatic systems, to simplify the production of visual optic models (**Figure 2**). By analyzing a range of fish species, he showed how refractive index adapts to different lens sizes (6) and analyzed cetacean eyes to demonstrate that secondarily aquatic mammals had convergently evolved the characteristic optics of fishes (**Figure 1**) (7). Another German scientist, Theodor Beer, showed that fish lenses accommodate through movement of the lens within the eye, since lens density prevents shape change as an accommodative mechanism (**Figure 2**) (8). These early studies provided a foundation for continued examination of fish lens optics.

**Figure 2:**
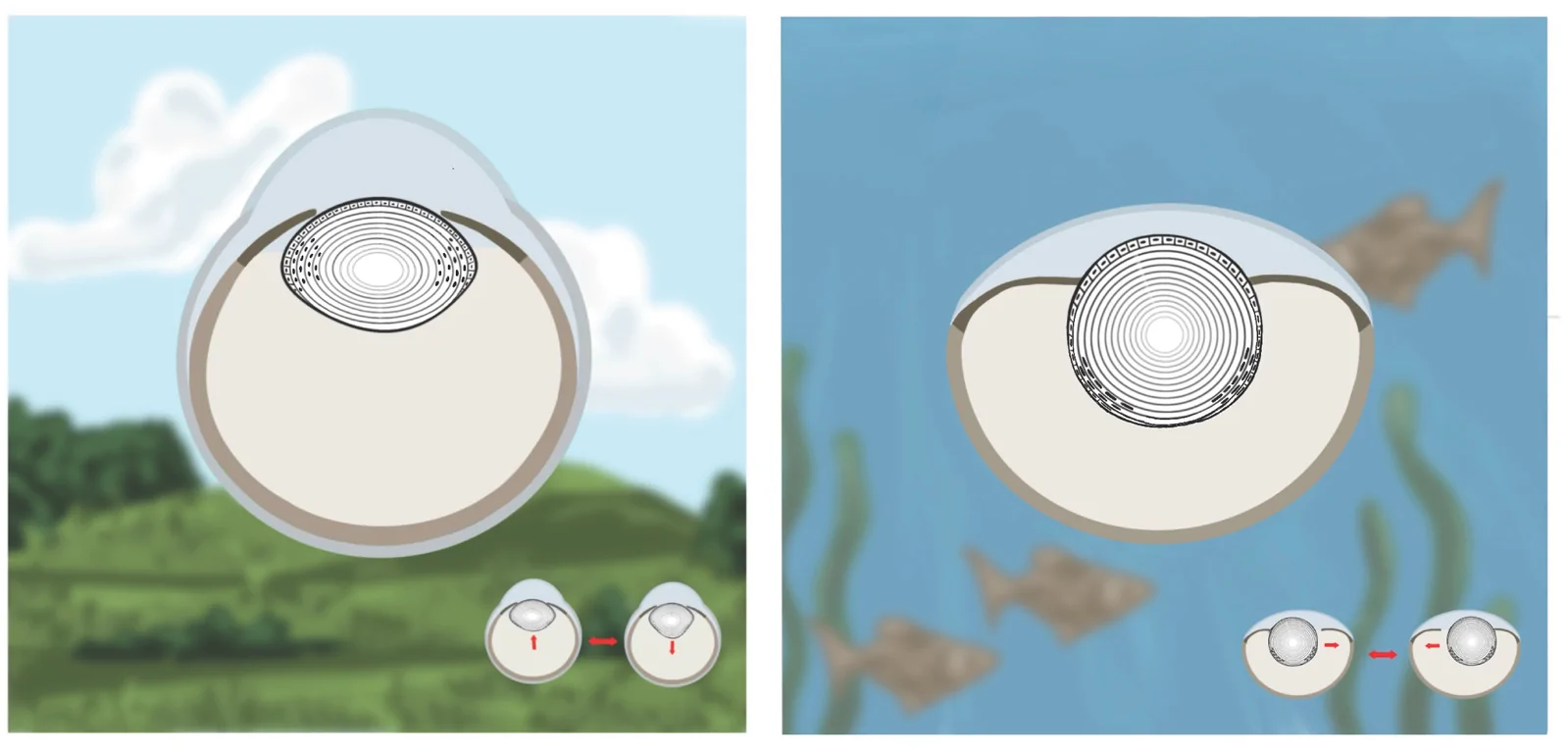
Comparison of the general structure of an aerial and aquatic eye. In aerial vision the cornea produces the greatest light refraction and the lens changes shape during accommodation. The typical aquatic eye relies on a spherical lens with higher protein concentration to provide appropriate refractive power, and accommodation occurs by moving the lens within the eye. The location of the transitional zone between outer epithelial and inner fiber cells is more posterior in fish species compared to mammals and the organelle free zone is wider. Illustration by Marin Dixon.

## Modern study of aquatic lens optics

While the spherical lens and non-refractive cornea of the fish eye simplifies mathematical modeling of visual optics, the details of these optics continued to be debated for over a century. Multiple authors since Matthiessen have both measured the lens GRIN and developed models to explain how it might alleviate spherical aberration, such as Sadler (9), Fernald and Wright (10), Jagger and Sands (11), and Kreuzer and Sivak (12) [reviewed in (13)]. Pierscionek provides excellent reviews of the different ways that a lens can produce a GRIN, how they are measured, and how they vary across aquatic and terrestrial lenses, and unanswered questions about GRIN formation (14, 15). Fish eyes typically have a short focal length relative to their large pupils to maximize light capture, leading to strong longitudinal chromatic aberration (16). A similar problem occurs in nocturnal terrestrial vertebrates. However, shallow-water fishes typically have strong color vision, making the presence of chromatic aberration a significant issue. This visual challenge is overcome with a multifocal lens that focuses different wavelengths of light at different planes (16). The optical properties of species’ lenses are adapted so that the prominent wavelengths in the environment are focused properly onto the photoreceptors (17). While the human lens is monofocal (16), multifocal lenses are widespread across both aquatic and terrestrial vertebrates, including other primate species (18). Analysis of a lens’ multifocal nature is simplified in aquatic species as the terrestrial cornea also produces spherical aberration, which must be adjusted for by the terrestrial lens (19). Pupil shape further impacts aberration as a slit-like pupil narrows the light column across the lens (18). Since most fish species lack an adjustable pupil, they are also simpler to model. Interestingly, large predatory fishes that hunt in open blue water, often at depths with low light levels, have monofocal lenses (20).

Studies on diverse aquatic species have detailed how lens optics change during development, and interestingly, how optics in the adult eye adjust as individuals move between different visual environments. The ability to easily image the zebrafish lens at all developmental stages illustrated how GRIN changes as the eye grows (21). Zebrafish studies also showed that the lens nucleus moves from the anterior pole to the center during larval development and that this movement is altered in mutants lacking one of the Aquaporin 0 paralogs, Aqp0a (22). This paralog, and not Aqp0b, was responsible for maintaining the anterior suture and repositioning of the lens nucleus, which are both required to maintain lens transparency. A similar central shift in lens position during development was recently identified in the clawed frog, *Xenopus laevis*, showing that it is not a unique feature of zebrafish or teleost fishes (23). The African cichlid *Haplochromis burtoni* relies heavily on color vision for social interactions, making it a good model to investigate the impact of visual inputs on lens development (**Figure 1**). Kröger et al. (24) found that individuals raised under monochromatic light developed lenses with altered gradient refractive index properties, although the mechanism was not established. While these studies establish optical flexibility during development, changes in an adult lens are also possible. Using another cichlid species, Schartau et al. (25) showed that the refractive properties of the lens within the organelle-free zone changed between the light-adapted and dark-adapted state. In the dark the lens became more monofocal, presumably adjusting to the use of rod-dominated vision. How organelle-free fiber cells are adjusting optics was not determined.

Aquatic species have contributed to the study of emmetropization, the regulation of eye growth to match the refractive power of the anterior segment of the eye. Myopia, a mismatch in refractive power and eye length that puts the visual focal plane short of the retina, is the most common visual disorder in humans (26). One approach to studying emmetropization is to apply eye covers that block vision or change focus to determine the impact on eye growth. Covering the eyes of the Nile tilapia (*Oreochromis niloticus*) with diffusers causing form deprivation myopia induced changes in eye length, showing that fishes respond similarly to birds and mammals by integrating visual information into the regulation of eye growth (27). Interestingly, this mechanism did not involve changes in the optics of the lens itself. More recently zebrafish have been developed as a model system for exploring the genetic mechanisms behind emmetropization. Optical coherence tomography has been used to quantify eye dimensions in zebrafish, providing a platform for testing the impact of myopia-related genes on eye growth (28) and multiple studies have identified genes of interest (29–34). New methods for monitoring the optical development of the zebrafish eye could be applied to the exploration of mechanisms regulating eye growth, with direct applications to understanding and treating human myopia (21).

Aquatic species provide unique opportunities to explore the evolution of optical systems functioning at the water’s surface. Perhaps most famously, central and south American species in the genus *Anableps*, also known as the four-eyed fishes, have eyes adapted to see simultaneously in water and air (35). Retinoscopic measurements through the dorsal and ventral pupil confirmed previous hypotheses that the different regions of the pear-shaped lens are optically adapted for either aerial or aquatic vision. The need for vision in both water and air is important in the multiple mammalian taxa that returned to aquatic environments. Cetaceans are the most aquatic of these groups, and the eyes of dolphins, porpoises, and whales have a thickened sclera and cornea and a flattened corneal shape compared to terrestrial mammals (36). The cetacean lens is spherical, and the ciliary muscle is poorly developed or absent, suggesting a lack of accommodation through change in lens shape (**Figure 1**). Instead, like in fishes, the eye likely accommodates through shift in lens position (37). The cetacean eye should be myopic during aerial vision, but various hypotheses have been proposed for how vision is corrected, including flattened regions of the cornea, use of different lens regions with varying refractive indices, or pupillary constriction (38, 39).

Pinnipeds, which includes seals, sea lions, and other close relatives, are more terrestrial than cetaceans. Work with live seals showed that their lenses are spherical or nearly spherical and that their flattened cornea contributes little refractive power in air (40). While they appear to have a strong ciliary muscle, little accommodation was found. Study of another seal species found strong evidence for a multifocal lens, which was surprising considering that this group is not thought to have color vision (41). Seals also show other visual adaptations to aquatic vision under varying light intensities, including a muscular iris capable of a wide range of pupil size, and a well-developed tapetum lucidum, a reflective layer behind the retina that allows light to pass a second time through the photoreceptive cells to increase light capture (42, 43). Many frog species face the different challenge of needing aquatic vision during their larval stages while shifting to aerial vision during metamorphosis into a terrestrial adult. Metamorphosis in species with terrestrial adults produces a flattened lens, while species that remain aquatic retain a more spherical lens (44). Understanding mechanisms that allow lens optics to change significantly during development or switch between different optic demands illustrates the adaptability of optical systems, providing possible insights into manipulation of eye focus with implications for treating myopia, a major cause of vision loss in humans.

Aquatic lenses from non-vertebrate species have also attracted attention. Members of the Cephalopoda include squids, octopuses, and cuttlefishes and contain an eye similar in structure and optics to the vertebrate camera eye (**Figure 1**). The squid eye optically corrects for spherical and chromatic aberration and accommodates using lens movement, similar to the teleost fish eye, but the lens is more flattened and tends towards hyperopia (45). As we will see below, the convergent evolution of a large camera eye within both vertebrates and invertebrates provided important insights into the evolution of lens proteins that provide high refractive index. Camera-style eyes in the larval stages of arthropod invertebrates have also been studied. Larval stages of common marine crustacean copepods in the genus *Copilia* have an eye that is essentially a single enlarged ommatidium from a compound eye with a corneal lens and crystalline cone (46). These copepod species attracted attention due to their unusual oscillating photosensitive rhabdom, which acts as a scanning device and may amplify sensitivity in low light conditions. The aquatic arthropod that has been most studied for its camera-style eye is the sunburst diving beetle, *Thermonectus marmoratus*. Larvae of this species are aquatic predators with 12 eyes, four of which are forward pointing and tubular, contain bifocal lenses, and scan the environment as the larva moves its head from side to side (47). This species is a good comparative model for eye lens biology, showing that unlike vertebrates, achievement of emmetropia does not require visual input during development, and that alteration of osmoregulation may produce enlargement of the eye while maintaining proper focus (48, 49). Comparing the physiological differences between this larval arthropod, cephalopod invertebrates, and vertebrates illustrates the range of evolutionary strategies leading to the development of a biological, transparent, refractive focusing element.

## The diversity of lens structural proteins

Aquatic vertebrates have played a key role in studies describing the diversity, evolution, and function of the highly expressed “crystallin” proteins that produce the refraction and transparency of the lens. Lens crystallin proteins are not only significant for their role in allowing the lens to transmit and focus light, but they provided the first model for describing gene co-option and gene sharing, since multiple families of lens proteins evolved from metabolic enzyme and stress response proteins used in non-lens tissues (50–52). Because fishes represent a wider evolutionary range of taxa living in a greater diversity of visual environments than terrestrial vertebrates, they have been a valuable comparative group for study of lens protein biochemistry. Early biochemical analyses of lens proteins from the 1950s and 1960s found that fish species contained all three ubiquitous lens crystallins: the α, β, and γ-crystallins (53–55). Subunit and amino acid analysis confirmed that α-crystallin orthologs also occurred in the dogfish shark and jawless lamprey (56). Zigler and Sidbury (57) identified α-, β-, and low molecular weight crystallins (presumably γ-crystallins) in the smooth dogfish lens (*Mustelus canis*). Interestingly, taxon specific crystallins such as the δ-crystallins of birds and other reptiles, ε-crystallin of frogs, and τ-crystallin in turtles (58) have not been found in fish species. However, fishes do express a diverse and abundant type of methionine rich γ-crystallin called the γM-crystallins, first identified in a catfish (59). The full diversity of fish γ-crystallins was more fully explored by Wistow et al. (60) as they recognized γN-crystallins, and by Kiss and Cheng (61) in the Antarctic toothfish (*Dissostichus mawsoni*).

Interestingly, Rabaey’s (62) analysis of 35 fish species showed a greater complexity of proteins and lower overall α-crystallin content, perhaps reflecting the presence of these diverse γM-crystallins and mirroring the lower α-crystallin concentrations later found in the zebrafish (63). Chiou et al. (64) published a biochemical analysis of lens crystallins from the carp (*Cyprinus carpio*) identifying all three ubiquitous families but noted that the high molecular weight fraction produced by gel exclusion chromatography, which is typically purified α-crystallin, was in this case a mix of both α- and β-crystallin. This mix of α- and β-crystallins was also found when separating lens proteins from the Antarctic toothfish (65). We found a similar co-elution of α- and β-crystallins when analyzing zebrafish lens proteins (66), suggesting an interaction between α- and β-crystallins in these fish lenses that does not occur in mammalian lenses. Various proteomic analyses have quantified lens crystallin content of the zebrafish lens, both in adults and throughout development (63, 66, 67). More recently, advanced mass spectrometry methods have been developed to quantify lower abundance proteins in the lens cortex and nucleus (68). Remarkably, this new approach was able to detect over 13,000 unique peptides and makes the small zebrafish lens a useful tissue for high-resolution lens proteomics.

One benefit of studying non-mammalian lenses is that species vary in body temperature and can be used to better understand thermal effects on crystallin function. McFall-Ngai and Horwitz (69) examined the ability of lenses from 12 vertebrate species ranging in physiological temperature from -2°C (*Trematomus bernacchii* from the Southern Ocean) to 47°C (*Dipsosaurus dorsalis* from the Mojave desert) to maintain transparency and soluble lens protein under increasing thermal stress. They found that lenses from species with higher preferred and maximal temperatures were better able to resist temperature-induced opacity and protein insolubility. Lens α-crystallins from these species were most resistant to heat-induced aggregation. Zebrafish α-crystallin tertiary structures are less thermostable than their human orthologs, matching their lower physiological temperature (70). Another comparative analysis cloned αA-crystallin genes from six fish species ranging in physiological extremes from -2°C to 40°C and produced recombinant protein from each (71). Protein function varied with physiological temperature and three specific amino acid substitutions were identified that altered either protein stability or protective chaperone-like activity. Computer modeling suggested that one of these substitutions, a valine to threonine substitution at residue 62 in the cold adapted Antarctic toothfish *Dissostichus mawsoni*, alters the surface hydrophobicity of αA-crystallin and destabilizes oligomer formation, possibly increasing subunit exchange to compensate for the lower physiological working temperatures. Proteins in the Antarctic toothfish lens have adapted both to cold temperatures and the long lifespan of this species. Surprisingly, a γS-crystallin from the toothfish is structurally stable up to 56-63°C, well above the -2°C physiological temperature of the species, providing a model for examining mechanisms behind protein stability (72).

While fish lenses appear to adapt to the upper temperature in a species’ physiological range to avoid heat-induced cataract, thermal effects are not as notable for cold-induced cataract. Fish lenses remain clear at temperatures 10-40°C lower than that of mammalian lenses, but the differences in cold tolerance between fish species can be narrow (-10 to -20°C) (73). While Ferguson et al. (74) showed that insoluble protein from fish cold-induced cataract was likely γ-crystallin, Loewenstein and Bettelheim argued that the opacity resulted not from differences in individual crystallins, but due to differences in the molecular arrangement of lens proteins. To support this conclusion, they noted that the boundary between the lens nucleus and cortex developed an irreversible cold cataract, which they argued was due to a different arrangement of lens crystallins as part of the positive birefringence produced in this boundary region. A more recent study did find a large difference in cold tolerance between lenses from a tropical fish and the Antarctic toothfish, with the latter remaining clear after a 48-hour incubation at -12°C, while the tropical fish developed nuclear opacity at 0°C, and suggested that adaptation in the fish’s γ-crystallins may account for this tolerance (65). A follow-up study detailed the molecular diversity of the Antarctic toothfish lens crystallins, identifying 14 γ-crystallins and arguing that their diversity allows for polydispersity leading to cold stability (61). A biophysical examination of six Antarctic toothfish γ-crystallins found that the liquid-liquid phase separation characterizing cold cataract was not related to thermal stability or chaperone activity, but instead a product of protein interactions through charged resides, providing a model system for discovering how protein stability at cold temperatures could be manipulated (75). This is another example of how naturally evolved lens crystallin proteins in diverse aquatic organisms provide insights into the design of protein stability.

The abundance of numerous γ-crystallins in fish lenses has provided a model system for exploring lens protein evolution. For example, Wistow et al. (60) used a phylogenetic study of aquatic and terrestrial β- and γ-crystallins to hypothesize that the γN-crystallins are a β/γ hybrid, bridging an evolutionary gap between the two crystallin families. As more chordate genomes are annotated, broad comparisons across the phylum have been used to examine the evolution of cysteine residues within lens crystallin proteins, which can lead to aggregation when oxidized (76). These comparisons show that cysteines are not conserved in α- and β-crystallins but are surprisingly abundant in the γ-crystallins, likely contributing to their ability to produce long-term lens transparency and high refractive power. Antarctic toothfish lens has been used to explore how their two γS-crystallins, both homologs to human γS-, have adapted to cold temperatures, while still maintaining the molecular characteristics required for high refractive index, providing a model for protein resistance to chemical and thermal denaturation (77). Even the non-vertebrate tunicate (*Ciona intestinalis*) has been useful in understanding the evolution and function of Ca^2+^ binding in β/γ-crystallin function (78).

While vertebrate lenses include co-opted stress proteins (α-crystallins) and house-keeping enzymes (δ- and τ-crystallins), the squid lens incorporates a co-opted glutathione S-transferase (S-crystallin), leading to the hypothesis that lens evolution benefited from the use of stress and enzyme-related genes (50). Squids such as *Loligo opalescens* have been a useful model for understanding lens crystallin protein function since they contain only one major crystallin type (S-crystallin) with 25 individual proteins descended from a single glutathione S-transferase ancestor (79). These proteins are expressed differentially across the squid lens and have been under positive selective pressure to alter their dimerization and electrostatic properties, illustrating how protein evolution is harnessed to produce an optically complicated eye lens (80).

Cephalopods also express two taxon-specific lens crystallins (Ω- and O-crystallin), and an L-crystallin related to Ω-crystallin in the lens of the light emitting squid photophore (Reviewed in (81). Another mollusk, the scallop, uses an unusual Ω-crystallin that forms a tetramer between ancestrally cytosolic and mitochondrial alcohol dehydrogenases (82). Beyond the Mollusca, the eyes of cnidarian cubomedusans have been well examined as they contribute to a rhopalium that the animal uses to navigate its environment (83). The species *Tripedalia cystophora* was used to identify three classes of lens crystallins. The J1 crystallins include a group of three similar gene products likely due to gene duplication events (84), while the single J3-crystallin is related to the multi-functional saposins (85). A proteomic analysis of eye tissue from the arthropod sunburst diving beetle larva *Thermonectus marmoratus* identified 10 lens proteins, with six having sequence homology to cuticular proteins, while others were similar to a serine protease and a protein involved in endoplasmic reticulum protein translocation (86). Reduction of the expression of one of these proteins using RNAi led to lens cataract, arguing for its functional importance (87). Altogether, the inclusion of diverse, aquatic species has provided a broader set of independent examples to support the hypothesis that eye lenses evolved by coopting genes involved in stress response, detoxification, or other enzymatic reactions (50). Aquatic lenses also supported an understanding of how changes in gene expression evolved to produce the high protein densities required for light refraction (58).

Because teleost fishes (a large taxon containing most fishes with a bony skeleton) underwent a whole genome duplication early in their evolution, genomes sometimes contain two copies (paralogs) of a single-copy mammalian gene. This was found to be the case with αB-crystallin after a second gene was identified in the zebrafish (88). While the mammalian αB-crystallin ortholog is expressed throughout the body, unlike the lens-preferred αA-crystallin, the expression of the two zebrafish αB-crystallin paralogs has diverged. In adults, the originally discovered paralog (now known as αBa-crystallin) shows lens-preferred expression, similar to zebrafish αA-crystallin (89, 90). The second paralog, however, shows the more widespread expression of mammalian αB-crystallin, with transcripts identified in adult brain, liver, cardiac and skeletal muscle, although the highest expression was found the lens (88). The lens-preferred αBa- expression of zebrafish adults is not found in early larval stages. At 4 days post fertilization (dpf) we found αBa-crystallin tryptic peptides by mass spectrometry parallel reaction monitoring in extraocular tissue, which fell to almost undetectable levels by 7 dpf (91). These results are mirrored by single cell-RNA seq data, which show very low *cryaba* transcript levels through 5 dpf, and highest expression in lateral line cells, followed by lens epithelium (92). By contrast, the other paralog, *cryabb*, was expressed at much higher levels, but exclusively outside of the eye, mostly in otic capsule, muscle, and hair cells. These data show that both zebrafish αB-crystallin paralogs undergo an ontogenetic shift in expression to produce the adult profile. However, the αA-crystallin gene *cryaa* shows lens-preferred expression from the earliest stages of development, being found almost exclusively in lens fiber cells starting at 2 dpf when its expression first increases.

The developmental roles of zebrafish α-crystallins both within and outside the lens are not known. While we found that CRISPR-induced homozygous loss of function of the *cryaa* gene led to subtle gross defects in the lens, damage to either αB-crystallin gene caused no noticeable lens defects at 3 or 4 dpf (93). However, another study showed a lens phenotype after disruption of either αB-crystallin gene at those same ages (94), even though it appears that *cryaba* lens expression at those ages is very low, and *cryabb* lens expression is almost non-existent (92, 95). Since the adult zebrafish lens expresses two abundant α-crystallins, it is possible that the protective roles of αA-crystallin are shared with the lens-preferred αBa-crystallin. We were interested to see how the lack of either protein might affect the development of cataracts as zebrafish age. Classic work on the mouse knockout lines for each mammalian α-crystallin showed that loss of the *Cryaa* gene led to early onset cataracts, but loss of *Cryab* did not (96, 97). Zebrafish have typical lifespans in a research facility of approximately 5 years, slightly longer than the mouse. We recently published the first data on age-related cataract prevalence in any strain of wildtype zebrafish, showing that approximately 27% of wildtype fish developed some amount of lens opacity by 18 months of age, with similar levels at 24 months (19%) (95). Loss of αA-crystallin did not increase these percentages, but interestingly, loss of αBa-crystallin did, doubling the cataract incidence at 24 months and producing the most severe examples of opacity. These data suggest that in the zebrafish αBa-crystallin may play an important role in stabilizing other lens proteins during aging to prevent aggregation, perhaps replacing αA-crystallin in this role.

One hypothesized function for α-crystallins in the lens is to act as chaperone-like proteins, binding to hydrophobic regions of denaturing proteins as they age and preventing them from becoming insoluble and leading to opacity (98). Using classic assays that measure an α-crystallin’s ability to prevent the aggregation of chemically denatured protein, we found that zebrafish αA-crystallin was a stronger chaperone than human αA-crystallin at temperatures from 25 to 40°C (70). However, when comparing protection at each species’ typical physiological temperature (28°C for zebrafish and 37°C for human), the zebrafish and human recombinant proteins showed similar protection, suggesting that the zebrafish ortholog is adapted to work at a lower body temperature. We also found that zebrafish αBa-crystallin produced weaker protection than human αB-crystallin and its zebrafish αBb paralog (88). We interpreted these differences between zebrafish αB-crystallin paralogs to suggest that the primary role of the lens specific αBa-crystallin was not protection against protein aggregation, but another, yet to be identified lens specific role. However, Koteiche et al. (99) used a different assay that measured the binding of crystallins to destabilized target proteins to show that it was in fact zebrafish αBa-crystallin that was the most effective chaperone, with stronger ability to bind proteins than the other two zebrafish α-crystallins. These data showing zebrafish αBa-crystallin’s stronger ability to bind destabilized protein fits with our data indicating that zebrafish lacking the *cryaba* gene were more prone to age-related cataract (95).

The presence of two αB-crystallin paralogs in zebrafish is likely the result of the genome duplication event that occurred at the base of teleost evolution. The fact that a second αB-crystallin paralog has not been found in non-teleost vertebrate taxa, including cartilaginous fishes and osteichthyan taxa closely related to teleosts, such as the gar and bowfin, suggests that the ancestral condition was to have one αB-crystallin and that mammals and other tetrapods simply retained this single protein (100, 101). The occurrence of paralogs is sometimes seen as a complication when using teleost fishes, such as the zebrafish, as model species. But as is the case with αB-crystallins, it instead provides opportunities to dissect the functions of multifunctional proteins. We will see that this approach has also been helpful in better understanding the function of aquaporins in lens physiology.

## Fish cataracts, lens physiology, and aging

Significant work from the 1950s through 1960s on lens resting potentials, ion permeability, and solute transport was done with frog species (102–104). However, Rae et al. (105) appear to be the first to measure the lens potential in a fish species, the rainbow trout, as a first step towards investigating the presence of cataract in hatchery raised individuals. Study of fish lens physiology was driven by interest in cataract etiology and prevention in commercially important species (106). An increase in the prevalence of cataracts in wild, non-hatchery raised salmon in Scotland was linked to the use of a chemical for the prevention of parasitic sea lice in neighboring farmed salmon (107). However, Dobson and Schuurman (108) took issue with that study’s design and its interpretation of past literature, instead stressing previous work suggesting other factors leading to fish cataract, including diet, UV light exposure, mechanical injury, and osmotic stress. A large increase in the prevalence of cataracts in European farmed salmon occurred after the removal of blood meal from their diets as a step to prevent the spread of bovine spongiform encephalopathy (109). Experimental feeding studies showed that diets supplemented with histidine could reduce the incidence of cataracts (110–112). In farmed rainbow trout (*Oncorhyncus mykiss*) parasite-induced cataracts were more prevalent in female fish and were correlated with delayed maturation (113). Multiple other factors have been linked to lens cataract in farmed fishes, such as increased oxidative stress in triploid fish populations, higher water temperature, and higher dissolved CO_2_ levels (114–116).

Another fish species has been developed as a cataract model due to its propensity for developing lens opacity. The common lumpfish (*Cyclopterus lumpus*) is used as a cleaner fish to remove parasitic sea lice from farmed salmon. Cataract prevalence in farmed lumpfish has been found as high as 100%, with a wild population at 33%, and many with severe cataract (117). Lumpfish cataracts occur as anterior opacities likely resulting from disruption of nuclear region fiber cells and anterior, subcapsular epithelial dysplasia (118). Cataract in farmed lumpfish is affected by diet (119) and lowering vitamin A levels in food led to reduced cataract and oxidative stress in the lens (120). Another fish species, the African turquoise killifish (*Nothobranchus furzeri*), is the shortest living vertebrate species that can be bred in captivity, with one strain exhibiting a median lifespan of only 4–6 months (121, 122). While the species has been used to examine age-related degeneration in the brain and retina (123), there is potential for studying lens aging as well. Because of its original use as a developmental model, the zebrafish has only recently gained attention as a model for age-related diseases (124–127). Considering the typical laboratory lifespan of 4–5 years (128), zebrafish may provide a valuable model going forward to study the causes and prevention of lens cataract, as well as other aging processes in the lens (95). The lower expense of housing zebrafish compared to rodent models makes them an attractive model for these aging studies.

The presence of gene duplicates in zebrafish has also contributed to novel findings in lens physiology. These zebrafish gene paralogs resulting from the teleost genome duplication often lead to subfunctionalization, where the roles of the single-copy homologous mammalian gene/protein have become separated into the two zebrafish copies (129). James Hall and his colleagues used the presence of two aquaporin 0 paralogs to dissect the various functions of the single mammalian version (130). They found that the Aqp0a paralog serves as the primary water channel, with the Aqp0b serving an unidentified function. Follow up studies showed that the water channel function of Aqp0a, but not Aqp0b, is required to rescue early larval cataract caused by morpholino knock down of either Aqp0a or Aqp0b (131). However, studies with the tetraploid Atlantic salmon (*Salmo salar*) and their homologous group of four Aqp0 channels found that a single amino acid substitution at residue 19 can alter the water permeability of Aqp0b proteins (132). The same study found that both Aqp0a and Aqp0b can permeate water *in vitro*, and the pH regulation of Aqp0 channels varies between the two zebrafish paralogs, with Aqp0a increasing water permeability in basic conditions, while Aqp0b increased water permeability in acidic conditions, like their mammalian AQP0 ortholog.

Analysis of CRISPR generated aquaporin 0 null zebrafish mutants led to the interesting observation that in juvenile wild-type individuals the lens nucleus is located towards the anterior of the lens and shifts to a central location with age (22). The genetic loss of Aqp0a prevents this centralization and leads to instability of the anterior suture between fiber cells resulting in anterior posterior opacity as fish age (22). The shift in lens nucleus position depends on the regulation of water permeability by Aqp0a (133). Although the maximum refractive index was reached in loss of function single or double Aqp0a/Aqp0b mutants, the normal shape of the gradient refractive index was disrupted (134). The lens-specific AQP0 of mammals is unusual within the aquaporin protein family for its auto-adhesion properties, which are thought to be important for lens fiber cell function (135). The two zebrafish Aqp0 paralogs differ in their auto-adhesion properties, with Aqp0b exhibiting stronger adhesion due to a single amino acid substitution compared to Aqp0a (136). Interestingly, Aqp0b mutant lenses were similar to wild-type lenses in transparency, lens nucleus localization, and refractive properties, suggesting that their auto-adhesion property is not essential for the lens, or may be compensated for by other molecular mechanisms (21, 22). In total, this series of studies illustrates how zebrafish paralogs provide a valuable tool for investigating lens protein function. The presence of paralogs for multiple proteins with known lens function provides ample opportunities to use this approach in the future (e.g. *crygn1*/*crygn2*; *cryaba*/*cryabb*, *sox1a*/*sox1b*, *myca*/*mycb*, *pabpc1a*/*pabpc1b*).

Comparative analyses that include fish species have provided insights into other aspects of lens structure and physiology. An examination of fiber cell size in nine different fish species, including zebrafish, found that fiber cell thickness was remarkably similar across lenses of different sizes, and that thickness did not change with radial position in the lens (137). These authors concluded that the uniformity in fiber cell thickness argues against the idea that removal of water increases refractive index in the deeper cells of the lens, as proposed by Bassnett and Costello (138). Mechanisms that may alter water or protein content of deep lying fiber cells to regulate gradient refractive index are still unknown, although it has been suggested that binding of water to lens crystallin proteins can alter refraction (139, 140). Comparative analyses of fish gene sequences have also been valuable in understanding the function of the intermediate filament proteins filensin (Bfsp1) and CP49/phakinin (Bfsp2), highlighting specific regions important for lens function (141, 142). The intermediate filament organizing protein lengsin is expressed in the zebrafish lens and morpholino knockdown resulted in defective secondary fiber cell differentiation, indicating a broadly conserved role in lens development (143). Similarities in lens development between fishes and mammals are well established, and aquatic species have played valuable and unique roles in understanding the amazing mechanisms that convert the simple cuboidal epithelium of the anterior surface of the lens to the specialized, elongated, organelle-free internal fiber cells, as explained next.

## How to make a lens

One of the strongest roles for aquatic species in lens biology is in understanding early development and tissue regeneration. While not exclusively aquatic, amphibian species played a key role in understanding lens development starting from the foundational work of Hans Spemann who first showed that removal of the optic vesicle prevented lens induction from the ectoderm (144, 145). Perhaps due to the availability of externally fertilized embryos the frog remained an important model for studying embryonic induction, including the interactions that lead to lens differentiation in the ectoderm (146, 147). Availability of embryos and larvae were key to the early use of zebrafish as a developmental model species (148, 149). Easter and Nicola (150) were the first to describe the early stages of zebrafish lens development, noting two differences from the mammalian pattern: first, delamination of the lens from the surface ectoderm instead of invagination, such that a hollow lens vesicle is never formed. Second, the central fiber cells are arranged randomly instead of the linear arrangement seen in mammalian lens development. Several laboratories carried out large-scale chemical and retroviral mutagenesis screens in zebrafish [reviewed by Fadool and Dowling (151)]. Both chemical (152–154) and retroviral (155) screens produced a handful of mutants with abnormal lenses, and four altered genes were identified in these mutants. Around this same time, synthetic RNA molecules called morpholinos were developed that could be microinjected into early-stage zebrafish embryos to block protein translation or alter mRNA splicing (156).

With both forward and reverse genetic tools in hand multiple labs began detailing the molecular control of zebrafish lens development (157). Novel gene targets for study were identified from forward screens while genes known to be important in mammalian lens development were also examined. For example, two papers cloned the transcription factor genes *foxe3* and *pitx3* already known to be involved in mammalian lens development and showed that they played similar roles in the zebrafish lens (158, 159). Morphants (animals in which protein translation was blocked with morpholinos) established that Pitx3 works upstream to potentially regulate *foxe3* expression (159). This early work began to establish conservation in lens development between zebrafish and mammals and highlighted the benefits of suppressing translation of specific transcripts in external zebrafish embryos. Furthermore, the presence of maternal protein and mRNA in early embryos allows for the study of homozygous recessive mutants that lead to lethality in mammal models (160).

In a pair of papers Greiling and Clark produced the first detailed cell fate maps for zebrafish lens development. Taking advantage of a zebrafish line with constitutive cell membrane fluorescence, they confirmed the formation of the lens through delamination of the lens placode and the differentiation of primary lens fibers as an irregular central mass (161). They also confirmed that secondary lens fiber cells differentiate from epithelial cells posterior to the equator, although the location of the epithelial proliferative zone remained unclear. Lastly, they identified separation of the lens and cornea between 19 and 24 hours post fertilization (hpf) without the presence of apoptosis, hypothesizing that separation involved changes in cell adhesion. In a follow up study Greiling, Aose, and Clark (162) fluorescently labelled individual lens placode cells to follow their fate as the lens developed. Here, they found that proliferation in the lens epithelium was widespread through 68 hpf, after which it became restricted to lateral regions. This study also found that central cells in the lens placode migrated to the posterior lens mass and differentiated into primary fiber cells, while lateral placode cells migrated to the anterior lens mass to become the lens epithelium. This pattern is similar to the mouse, and the authors used this as evidence to suggest that molecular control of lens differentiation in mouse and zebrafish are conserved. Injection of GFP-tagged αA-crystallin gene promoter regions showed that both mouse and zebrafish sequences drove similar expression in the lens, again showing conservation of the mechanisms underlying lens formation (91).

The zebrafish has recently played a pivotal role in uncovering a novel mechanism in vertebrate lens development. The absence of organelles in lens fiber cells was recognized in the late 1800s, with the details of their loss first described by confocal microscopy of chicken lens sections, showing the rapid and coordinated removal of mitochondria and nuclei (163). While many studies have detailed the mechanisms behind the production of the lens organelle-free zone [(OFZ: reviewed in (164, 165)], there has been recent debate about the role played by autophagy. The presence of autophagic vacuoles in differentiating lens fiber cells was first described in rat lens organ culture using electron microscopy (166). Confocal microscopy identified fluorescently marked autophagosomes in epithelial cells and both primary and secondary differentiating fiber cells in a transgenic mouse line (167, 168). Thin section electron microscopy and immunolabelling of human and embryonic chick lens identified autophagosomes containing mitochondria in lens epithelial cells and differentiating fiber cells, suggesting an important role for mitophagy in organelle clearance during lens development (169). However, mice lacking *Atg5*, a gene required for most types of autophagy, undergo normal fiber cell organelle loss (168, 170). Overall, these studies suggest that while autophagy plays an important role in lens cells, it is not sufficient to clear organelles from differentiating fiber cells. A CRISPR Cas9 knockout analysis of zebrafish genes with highly expressed lens transcripts showed that the phospholipase gene *plaat1* was required for fiber cell organelle removal during lens development (171). Follow up experiments showed that similar retention of lens fiber cell organelles occurred with knockout of the mouse Plaat3 gene, demonstrating the conserved nature of this developmental mechanism. Experiments in stable null lines for each gene in zebrafish and mouse detailed a mechanism that targets Plaat1 and PLAAT3 proteins to organelle membranes under the regulation of Hsf4/HSF4. This study exemplifies the utility of zebrafish as a high-throughput gene screening system, with the potential for follow up mechanistic studies in stable lines and other model organisms, such as mouse. Genomic comparisons across taxa can also provide insight into a gene’s importance, as a recent comparative study of 311 vertebrate species found greater divergence in Plaat1 coding sequences in species with low visual acuity, such as cave-dwelling species and the Asian swamp eel (172).

The zebrafish will continue to be a valuable tool going forward for detailing mechanisms of fiber cell differentiation. Multiple studies have shown that zebrafish lens fiber cell denucleation is complete by 72 hpf (161, 162, 173, 174) and requires DNase1l1l under the regulation of Hsf4 (175). There are limited studies examining the timing of mitochondria, endoplasmic reticulum (ER), and Golgi apparatus loss during zebrafish lens development. A study examining a zebrafish mutant for one of the lens specific connexins (*gja8b*) used immunohistochemistry to stain the ER marker protein KDEL and showed ER clearance from central fiber cells by 72 hpf, with ER retention in the mutant (176). The most comprehensive examination of organelle loss during zebrafish lens development comes from Morishita et al. (171) as they identified the role of Plaat1 in organelle degradation. That study used stable and transiently expressed fluorescent protein tags to show that mitochondrial and ER clearance spreads from the center of the lens outwards starting between 55 and 61 hpf, with the OFZ expanding to nearly 75% of the lens diameter by 96 hpf. It is notable that this OFZ appears narrower than the nucleus free zone by that stage, suggesting different timing for the removal of nuclei and other organelles. The retention of a narrow nucleated fiber cell zone, unlike the presence of nuclei deeper into the mouse lens, persists in the adult zebrafish lens (173). It is possible that the greater width of the zebrafish OFZ allows for a wider path of light through the lens as zebrafish do not use pupillary movement to narrow incoming light, meaning that a wider region of the lens must be transparent to prevent light scatter. Regardless of the functional consequence, the variation in width of the OFZ between mouse and zebrafish could provide a comparative approach to characterize the regulation of organelle loss. Understanding organelle loss has broad significance as keratinocytes and erythrocytes also remove organelles during their differentiation, and organelle loss is a ubiquitous aspect of cellular quality control. The loss of organelles in lens fiber cells provides a unique opportunity to explore the cellular control behind this important mechanism.

## Omics approaches and gene editing

Proteomic approaches have been used to compare developmental shifts in lens protein content between mammals and zebrafish. The first use of tandem mass spectrometry to quantify zebrafish lens proteins from 4.5 days to 6 months of age showed a shift in relative abundance during development, with various β-crystallins expressed throughout, while α-crystallins became more prevalent at 6 weeks (67). This study also identified several novel β- and γ-crystallins. A later study used 2D SDS-PAGE and MALDI-TOF analysis of lenses from 4.5 days to 27 months, noting similar low expression of α-crystallins through 6 weeks of age, increasing to around 6% of total protein in the adult lens, and several shifts in crystallin expression during development (66). Interestingly, this study found several similarities between zebrafish and mammalian lens development, including the delayed expression of γS-crystallin, early expression and later decrease in βB1-crystallin, and increased phosphorylation of αA-crystallin with age. Improved proteomic approaches were used recently to quantify over 13,000 proteins from adult zebrafish lens, analyzing abundance separately in the lens cortex and nucleus (68). This depth of identification allowed, for the first time, analysis of proteins beyond the highly expressed crystallins, and will be a valuable tool going forward to quantify changes in the proteome during development, aging, and disease.

Transcriptomic approaches often dominate studies of lens development and disease (177). They have been used regularly in studies with zebrafish and other aquatic species. For example, zebrafish morphants were used to show that a paralog of the eukaryotic translation initiation factor 3 (*eif3ha*) regulates translation of lens γ-crystallins (178), supporting the idea that lens crystallins are regulated both transcriptionally and translationally (179). RNA-Seq approaches have been used to study alterations in gene expression in various zebrafish lens-related mutants and to explore genetic regulation of optic fissure closure (180–185). The first single-cell transcriptomic atlas for vertebrate lens was generated with zebrafish, confirming the lens expression of many of the known regulators of mammalian lens development (92). Multiple single cell RNA-Seq analyses of human and rodent lens tissues have followed (186–189). Single cell transcriptomic analysis has been valuable in detailing the spatiotemporal expression of the three zebrafish α-crystallin genes and their possible roles in early lens development, showing the very low expression of both αB-crystallin genes (*cryaba* and *cryabb*) through 6 dpf in the lens, and that the single αA-crystallin gene (*cryaa*) is expressed almost exclusively in lens fiber cells (95). Multiomics approaches that combine single-cell and single-nucleus RNA seq with ATAC-seq and CUT&RUN-seq have provided a dynamic, developmental atlas of transcriptome and chromatin accessibility changes during lens development (190). While this study was done in chicken and mouse, similar approaches have been used across vertebrate species, including zebrafish, to examine retinal aging (191). Future work will likely extend the impact of multiomic approaches to lens studies in zebrafish and other aquatic species.

Another benefit of modern transcriptomic approaches is to identify genes with lens preferred expression that have no known lens function. Reverse genetics approaches can then mutagenize these genes and look for defects in lens development (192). Work with the mouse has produced rich datasets of developmental lens gene expression and alternate splicing (177, 193). These studies identified multiple RNA-binding protein genes that subsequent work validated as important, novel genes in lens development (193–195). Increasingly, these validation studies include CRISPR-directed gene targeting in zebrafish due to the short amount of time required to screen for and characterize phenotypes in crispant larvae. In conjunction with work in mammalian models, these zebrafish screens highlight conservation of function across vertebrate taxa (196). Our single-cell RNA seq atlas of zebrafish lens development highlighted 16 genes with preferred gene expression but with no known lens function, including six RNA-binding protein genes (92). We are currently screening these genes for roles in lens development and further analyzing our scRNA-Seq dataset to identify shifts in gene expression as lenses undergo fiber cell differentiation from 2 to 6 dpf. The zebrafish will continue to play a prominent role in discovering and detailing the molecular control of vertebrate eye lens development.

## Lens regeneration and degeneration

The ability of a newt to regenerate a removed or damaged lens was noted as early as the 1880s (Reviewed in (197)). This regenerative capacity is largely restricted to salamanders and frogs, although there are descriptions of lens regeneration in cobitid fishes and during a small window in chick development. In adult newts the new lens develops from dorsal iris pigment epithelial cells that undergo dedifferentiation to become new cell types (**Figure 1**). A significant sequence of papers from the 1960s and 1970s described this process, with details about molecular control published in the 1990’s. Amazingly, the ability to produce a normal, regenerated lens does not diminish after multiple rounds of damage and involves a robust and unchanging transcriptional profile (198, 199). Transcriptomic tools have characterized genes involved in regeneration (200–202), and optical coherence tomography has allowed visualization of lens regeneration in live animals (203, 204). Expanding molecular tools in the newt, such as transgenic reporter lines, allows for *in vivo* tracing of specific cell types, recently leading to the discovery that newt lens regeneration is facilitated by macrophages (205).

The African clawed frog *Xenopus laevis* is a commonly used developmental model species that has also been valuable for the study of eye tissue regeneration (206). Lens regeneration in this species differs from newt as the lens regenerates from corneal epithelial cells (207). While it was initially thought that *Xenopus* lens regrowth was restricted to larval stages and lost after metamorphosis, it has been shown to occur in post-metamorphic animals (208, 209). Because eye development in *X. laevis* is so well understood, the species has also been used to distinguish between mechanisms leading to initial development and regeneration, showing that apoptosis is required for regeneration alone (210). Lens regeneration in the axolotl (*Ambystoma mexicanum*), a charismatic paedomorphic salamander, was shown to be possible in a very narrow larval developmental range of 2–3 weeks (211). Unlike the newt, this regeneration takes place from both the dorsal and ventral iris. Gene expression analysis showed differences in the regeneration-competent iris and aged irises, suggesting genes that may be key to lens regeneration (212). Studies on lens regeneration in amphibians, like parallel work on retinal regeneration in zebrafish, takes advantage of powerful systems to examine the mechanisms of tissue transdifferentiation and repair, with promise to uncover molecular networks that could be manipulated in treatment of human disease. Insights from non-mammalian lens regeneration are informing early steps on the development of human lens regeneration (213).

Another aquatic species, the Mexican tetra *Astyanax mexicanus*, has provided unique insights into developmental connections between the lens and retina. This species has surface dwelling populations with normal vision and multiple subterranean populations with degenerate eyes that have evolved over the past 10,000 years (214). Lens loss is coupled with extensive apoptosis and reduction in *pax6* expression. Remarkably, transplantation of a surface fish lens into the eye of a cave fish during embryogenesis restored normal eye development, showing that signals from the lens could sustain retinal development (215). This same model system showed that increased *sonic hedgehog* expression contributed to eye degeneration (216) and that maintaining normal eye development may require interaction between the lens and retinal pigmented epithelium (217). The Mexican tetra is a potential model for understanding the signals that lead to, and could slow, retinal degeneration.

## A role for the aquatic lens going forward

The major emphases of eye lens research have shifted over the past 200 years. The eyes of aquatic species have played a significant role in understanding the physics of light refraction and focus, the study of protein evolution, lens cell structure, and the physiology that maintains lens transparency and adjusts refractive index. Besides the value of a broader diversity of taxa for comparative study, the different optical challenges faced by species living in water have set up a natural experiment to test the ways that optical systems adjust to different demands. Inclusion of more species across diverse taxa increases the sample size comparative biologists draw on for insights into lens evolution, development, and function.

The examination of diverse species facing different visual challenges helps to define the variation in possible optical systems. For example, species have evolved a range of approaches for using a lens and accommodative mechanisms to solve the fundamental problem of focusing images on the retina. While not addressed in this review, these optical variations are accompanied by plasticity in the structure and function of the retina as well. At the molecular level, many aspects of protein function remain conserved, but divergence in others, such as a switch in the specific DNase used during fiber cell differentiation or the subfunctionalization between protein paralogs in teleost fishes, informs our understanding of lens biology. Going forward, exploring the range of possibilities in lens function will guide the discovery of ways that human lens biology can be manipulated in the design of treatments for human disease.

Finally, aquatic species will continue to play unique roles in a multitude of other areas. The growth of the zebrafish as a developmental model species with the benefit of high throughput, cost effective genetic screening, makes it a likely contributor to future discoveries in lens development. Zebrafish is also growing as an aging model and can contribute to the study of age-related diseases such as cataract and myopia. Aquatic species will also continue to play a valuable role in regeneration studies. The continuing growth of -omics approaches, and annotation of more species’ genomes, will allow for greater use of epigenomics, transcriptomics, and proteomics approaches in the comparative study of lens biology. While aquatic species have played important roles throughout the different historical aspects of lens study, they will continue to provide value as cost-effective and efficient comparative species for the future of our discipline.
